
JOURNAL INFORMATION
==============================
NLM Title Abbreviation: CPT Pharmacometrics Syst Pharmacol
Journal ID: CPT Pharmacometrics Syst Pharmacol
NLM Journal ID: 101580011
Journal ID: PSP4

CPT: Pharmacometrics & Systems Pharmacology

EISSN: 2163-8306
Publisher: Wiley

ARTICLE INFORMATION
==============================
PMCID: PMC11474920
DOI: 10.1002/psp4.12987
Article ID: PSP412987
Article version: 1
Subjects: Issue Information, Issue Information

Issue Information

Electronic publication date: 2024 Apr 12
Publication date: 2024 Apr
Volume: 13
Issue: 4
First page: 505
Last page: 508
Copyright: © 2024 ASCPT
License: This is an open access article under the terms of the http://creativecommons.org/licenses/by/4.0/ License, which permits use, distribution and reproduction in any medium, provided the original work is properly cited.
License URL: https://creativecommons.org/licenses/by/4.0/

Figure count: 0
Table count: 0
Page count: 3

source-schema-version-number: 2.0
cover-date: April 2024
details-of-publishers-convertor: Converter:WILEY_ML3GV2_TO_JATSPMC version:6.4.9 mode:remove_FC converted:15.10.2024

==============================

An Official Journal of ASCPT and ISoP

### CPT: Pharmacometrics & Systems Pharmacology

Open Access

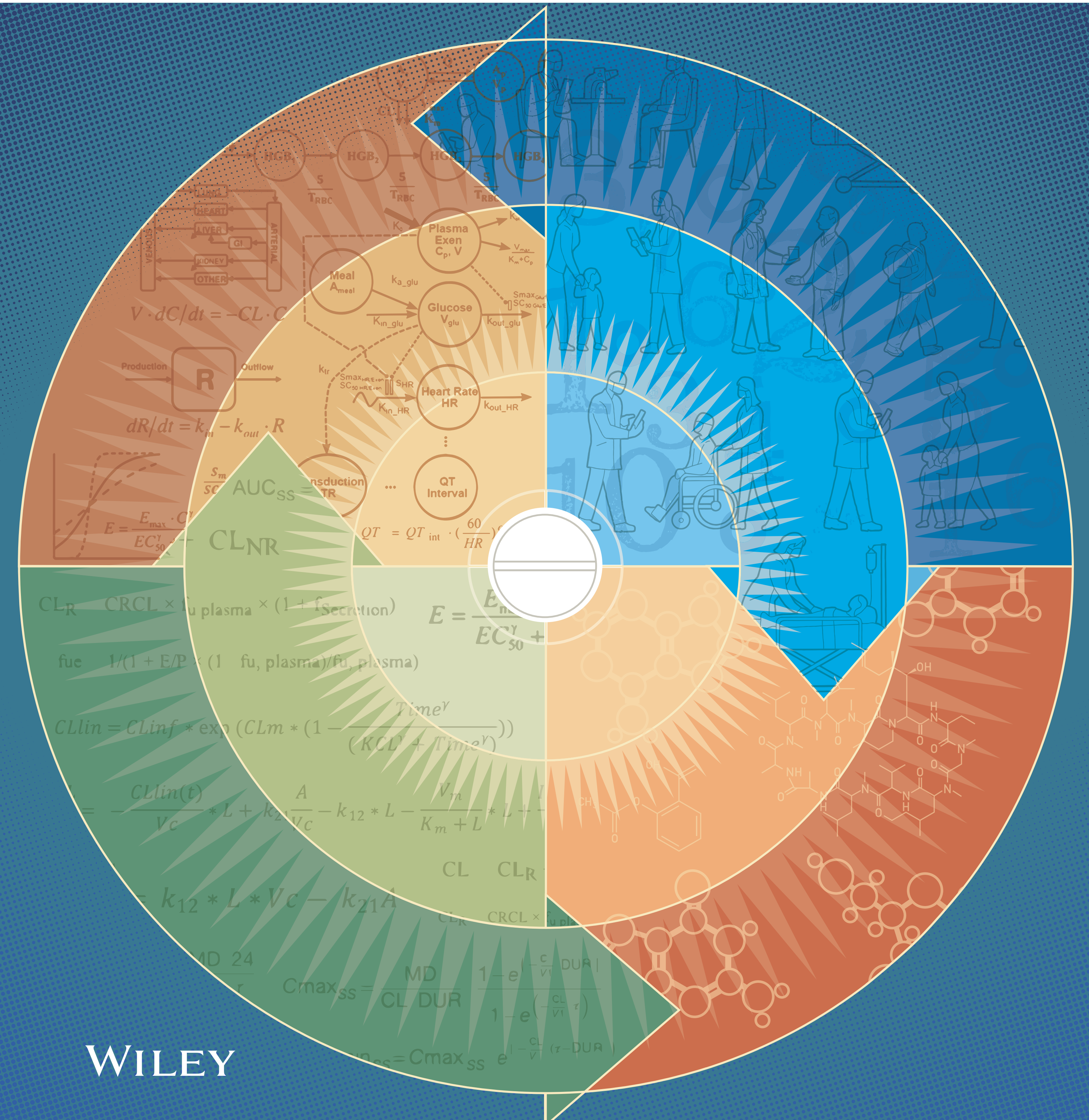

WILEY

### CPT: Pharmacometrics & Systems Pharmacology

WILEY

### Editor-in-Chief

France Mentré, MD, PhD, *School of Medicine of University Paris Diderot, Paris, France*

### Deputy Editor-in-Chief

Karen Rowland Yeo, PhD, *Certara UK Ltd., Sheffield, UK*

### Associate Editors

Paolo Denti, PhD, *University of Cape Town, Cape Town, South Africa*
Rajat Desikan, PhD, *GSK, Stevenage, UK*
Jonathan French, ScD, *Johnson & Johnson Innovative Medicine, Spring House, PA, USA*
Stefanie Hennig, MSc, PhD, *Certara Strategic Consulting, Brisbane, Australia*
Priya Jayachandran, PharmD, *Regeneron Pharmaceuticals, Inc., San Francisco, CA, USA*
Andrew Hooker, PhD, *Uppsala University, Uppsala, Sweden*
Sriram Krishnaswami, PhD, *Pfizer, Groton, CT, USA*
Ping Zhao, PhD, *Bill & Melinda Gates Foundation, Seattle, WA, USA*

### Editors-in-Training

Brian Cicali, PhD, *University of Florida, College of Pharmacy, Orlando, FL, USA*
Jane Knöchel, PhD, *AstraZeneca , Mölndal, Sweden*

### American Society for Clinical Pharmacology and Therapeutics

***Chief Executive Officer***
Abigail W. Gorman, *Alexandria, VA*

***Chief Operating Officer***
Elise S. Laffman-Johnson, *Alexandria, VA*

***Managing Editor***
Alethea B. Gerding, *Alexandria, VA*

***Deputy Managing Editor***
Alaina Webster, *Alexandria, VA*

***Senior Editorial Coordinator***
Aimee Greene, *Alexandria, VA*

### Editorial Board

Vivaswath S. Ayyar, PhD, *Janssen Research & Development, Spring House, PA*
Robert Bies, PharmD, PhD, *State University of New York at Buffalo, Buffalo, NY, USA*
Marc Birtwistle, PhD, *Clemson University Clemson, SC, USA*
Yuan Chen, PhD, *Genentech South San Francisco, CA, USA*
Marylore Chenel, PharmD, PhD, *Pharmetheus Uppsala, Sweden*
Emmanuel Chigutsa, PhD, *Eli Lilly and Company, Indianapolis, IN, USA*
Teresa Dalla Costa, PhD, *Federal University of Rio Grande do Sul - UFRGS Porto Alegre, Brazil*
Narendra M Dixit, PhD, *Indian Institute of Science, Bengaluru, India*
Anne-Gaelle Dosne, PharmD, PhD, *Janssen Research & Development, Beerse, Belgium*
Thomas Dumortier, Eng, MSc, *Novartis Pharma AG, Basel, Switzerland*
James M. Gallo, PharmD, PhD, *University at Buffalo, Buffalo, NY, USA*
Wei Gao, PhD, *EMD Serono, Billerica, MA, USA*
Jérémie Guedj, PhD, *INSERM, Paris, France*
Manish Gupta, PhD, FCP, *GenMab; Princeton, NJ, USA*
Bart Hendriks, PhD, *Novartis Institutes for BioMedical Research Cambridge, MA, USA*
Nick Holford, MBChB, *The University of Auckland Auckland, New Zealand*
Brett A. Howell, PhD, *DILIsym Services Division, Simulations Plus, Inc., Research Triangle Park, NC, USA*
Manuel Ibarra, PhD, *Universidad de la República, Montevideo, Uruguay*
Siv Jönsson, PhD, *Uppsala University Sweden, Uppsala, Sweden*
Kristin Karlsson, PhD, *Uppsala University Uppsala, Sweden*
Helen Kastrissios, PhD, *QuanTx Consulting, Brisbane, Australia*
Ron Keizer, PhD, *InsightRX San Francisco, CA, USA*
Steven E. Kern, PhD, *Global Health Labs, Bellevue, WA, USA*
Akash Khandelwal PhD, *UCB Biosciences GmbH, Monheim am Rhein, Germany*
Andrzej Kierzek, PhD, *Certara QSP; Sheffield, United Kingdom*
Qi Liu, PhD, *US Food and Drug Administration, Silver Spring, MD*
James Lu, PhD, *Genentech, Inc., South San Francisco, CA, USA*
Rajanikanth (Raj) Madabushi, PhD, *US Food and Drug Administration, Silver Spring, MD*
Efthymios Manolis, MSc, *European Medicines Agency London, United Kingdom*
Samer Mouksassi, PharmD, PhD, *Certara, Cairo, Egypt*
Jackson K. Mukonzo, PhD, *Makerere University, Kampala Uganda*
Joakim Nyberg, PhD, *Pharmetheus, Uppsala, Sweden*
Elsje Pienaar, PhD, *Weldon School of Biomedical Engineering, Purdue University, West Lafayette, IN, USA*
Daniel Scholes Rosenbloom, PhD, *Merck & Co., Rahway, NJ, USA*
Joe Standing, PhD, *University College London London, United Kingdom*
Natasha Strydom, PhD, *Amgen, Inc., San Francisco, CA, USA*
Yuichi Sugiyama, PhD, *Riken Innovation Center Sugiyama Laboratory Yokohama, Japan*
Elin Svensson, PhD, *Uppsala University, Uppsala, Sweden*
Joel Tarning, PhD, *Mahidol-Oxford Tropical Medicine Research Unit Bangkok, Thailand*
Nadia Terranova, PhD, *Merck KGaA, Lausanne, Switzerland*
Michael Tortorici, PharmD, PhD, *Aro Biotherapeutics, Philadelphia, PA, USA*
Coen van Hasselt, PhD, *Leiden University, Leiden, the Netherlands*
Paolo Vicini, PhD, MBA, *Confo Therapeutics, Ghent, Belgium*
Camille Vong, PhD, *Novartis AG Pharma, Basel, Switzerland*
Xinyuan (Susie) Zhang, PhD, *Daiichi Sankyo, Inc. Basking Ridge, NJ, USA*

### Overview

*CPT: Pharmacometrics & Systems Pharmacology* (PSP) is an official open access journal of the American Society for Clinical Pharmacology and Therapeutics (ASCPT) and International Society of Pharmacometrics (ISoP).

*CPT: Pharmacometrics & Systems Pharmacology* is a cross-disciplinary journal devoted to publishing advances in quantitative (e.g., modeling and simulation) methods as applied in pharmacology, physiology and therapeutics in humans. The journal welcomes original research articles, reviews and tutorials that bridge the following areas: pharmacometrics, modeling and simulation as applied to the design and evaluation of clinical trials, systems pharmacology modeling, particularly with a mechanistic link to human (patho)physiology, disease modeling, "population" or mixed-effects pharmacokinetics and pharmacodynamics (PKPD) modeling, modeling and simulation to support translational research, physiologically-based pharmacokinetics (PBPK), model-based meta-analyses of clinical trials, mechanism-based pharmacokinetic-pharmacodynamic modeling, computational pharmacology, bioinformatics, comparative efficacy, effectiveness and cost-effectiveness. Systems pharmacology may involve the application of systems biology approaches to study drug activities, targets and effects. The discipline is often defined with reference to engineering and pharmacological principles as the quantitative analysis of the dynamic interactions between drugs and a biologic system that aims to understand the behavior of the system as a whole. The common focus will be on quantitative methods that improve our understanding of pharmacology and therapeutics in humans.

**Abstracting and Indexing**
- Embase (Elsevier)
- SCOPUS (Elsevier)
- MEDLINE/PubMed indexing

**Society Information**
The American Society for Clinical Pharmacology and Therapeutics (ASCPT) was founded in 1900, and consists of over 2,200 professionals whose primary interest is to advance the science and practice of clinical pharmacology and translational medicine for the therapeutic benefit of patients and society. ASCPT is the largest scientific and professional organization serving the disciplines of Clinical Pharmacology and Translational Medicine.

**All articles published in this journal are freely available to read, download, and share. Articles are published under the terms of the Creative Commons Attribution Non-Commercial License which permits use, distribution, and reproduction in any medium, provided the original work is properly cited and is not used for commercial purposes.**

**Disclaimer**
The Publisher, the American Society for Clinical Pharmacology and Therapeutics, the International Society of Pharmacometrics, and Editors cannot be held responsible for any errors in or any consequences arising from the use of information contained in this journal. The views and opinions expressed do not necessarily reflect those of the Publisher, the American Society for Clinical Pharmacology and Therapeutics, the International Society of Pharmacometrics or Editors, neither does the publication of advertisements constitute any endorsement by the Publisher, the American Society for Clinical Pharmacology and Therapeutics, the International Society of Pharmacometrics, Editors, or Authors of the products advertised.

**Publisher**
*CPT: Pharmacometrics & Systems Pharmacology* is published by Wiley Periodicals, LLC, 101 Station Landing, Suite 300, Medford, MA 02155; Telephone: 781-388-8200; Fax: 781-388-8210.

### CONTENTS

CPT: Pharmacometrics & Systems Pharmacology

### PERSPECTIVES

### LETTER TO THE EDITOR

**509** Development of a cystic fibrosis population model for physiologically based pharmacokinetic models: Is it worth it?
**Eunjin Hong and Paul M. Beringer**

**REPLY**

**511** Response to "Development of a cystic fibrosis population model for physiologically based pharmacokinetic models: Is it worth it?"
**Magali Amiel**

### TUTORIALS

### TUTORIALS

**513** Application of multistate modeling to clinical data analysis
**Joachim Grevel, Joshua Veasy and Blesson Chacko**

**524** In silico modeling and simulation of organ-on-a-chip systems to support data analysis and a priori experimental design
**Nicoló Milani, Neil Parrott, Aleksandra Galetin, Stephen Fowler and Michael Gertz**

### RESEARCH

### CASE REPORT

**544** Simulation-based evaluation of personalized dosing approaches for anti-FGFR/KLB bispecific antibody fazpilodemab
**Kenta Yoshida, Victor Poon, Ajit Dash, Rebecca Kunder, Leslie Chinn and Matts Kågedal**

**ARTICLES**

**551** Population pharmacokinetic modeling of oral brepocitinib in healthy volunteers and patients with immuno-inflammatory diseases
**Farzaneh Maleki, Elias Clark, Christopher Banfield, Wonkyung Byon and Timothy Nicholas**

**563** Physiologically-based pharmacokinetic modeling of prominent oral contraceptive agents and applications in drug–drug interactions
**Gareth J. Lewis, Deepak Ahire and Kunal S. Taskar**

**576** Maximum likelihood estimation of renal transporter ontogeny profiles for pediatric PBPK modeling
**J. Porter Hunt, Samuel Dubinsky, Autumn M. McKnite, Kit Wun Kathy Cheung, Bianca D. van Groen, Kathleen M. Giacomini, Saskia N. de Wildt, Andrea N. Edginton and Kevin M. Watt**

**589** Physiologically-based pharmacokinetic modeling of the drug–drug interaction between ivacaftor and lefamulin in cystic fibrosis patients
**Magali Amiel, Alice Ke, Steven P. Gelone, Hannah M. Jones and Wolfgang Wicha**

**599** Tofacitinib pharmacokinetics in children and adolescents with juvenile idiopathic arthritis
**Cheng Chang, Camille Vong, Xiaoxing Wang, Anasuya Hazra, Annette Diehl, Timothy Nicholas and Arnab Mukherjee**

**612** Quantitative modeling of tumor dynamics and development of drug resistance in non-small cell lung cancer patients treated with erlotinib
**Anyue Yin, G. D. Marijn Veerman, Johan G. C. van Hasselt, Christi M. J. Steendam, Hendrikus Jan Dubbink, Henk-Jan Guchelaar, Lena E. Friberg, Anne-Marie C. Dingemans, Ron H. J. Mathijssen and Dirk Jan A. R. Moes**

**624** Evaluation of the drug–drug interaction potential of brigatinib using a physiologically-based pharmacokinetic modeling approach
**Michael J. Hanley, Karen Rowland Yeo, Meera Tugnait, Shinji Iwasaki, Narayana Narasimhan, Pingkuan Zhang, Karthik Venkatakrishnan and Neeraj Gupta**

**638** Pharmacokinetic/pharmacodynamic modeling of cortical dopamine concentrations after quetiapine lipid core nanocapsules administration to schizophrenia phenotyped rats
**Bruna Bernar Dias, Fernando Carreño, Victória Etges Helfer, Laura Ben Olivo, Keli Jaqueline Staudt, Karina Paese, Fabiano Barreto, Fabíola Schons Meyer, Ana Paula Herrmann, Sílvia Stanisçuaski Guterres, Stela Maris Kuze Rates, Bibiana Verlindo de Araújo, Iñaki F. Trocóniz and Teresa Dalla Costa**

**649** Data-driven disease progression model of Parkinson's disease and effect of sex and genetic variants
**Ryota Jin, Hideki Yoshioka, Hiromi Sato and Akihiro Hisaka**

**660** Understanding CYP3A4 and P-gp mediated drug–drug interactions through PBPK modeling – Case example of pralsetinib
**Christine Bowman, Michael Dolton, Fang Ma, Sravanthi Cheeti, Denison Kuruvilla, Rucha Sane, Nastya Kassir and Yuan Chen**

**673** A systematic efficacy analysis of tuberculosis treatment with BPaL-containing regimens using a multiscale modeling approach
**Maral Budak, Laura E. Via, Danielle M. Weiner, Clifton E. Barry III, Pariksheet Nanda, Gabrielle Michael, Khisimuzi Mdluli and Denise Kirschner**
